# Optimizing cerebrovascular endothelial health through shear stress modulation

**DOI:** 10.1113/EP092668

**Published:** 2025-12-08

**Authors:** Erika Iwamoto, Rintaro Sakamoto, Darren P. Casey

**Affiliations:** ^1^ School of Health Sciences Sapporo Medical University Sapporo Japan; ^2^ Human Informatics and Interaction Research Institute National Institute of Advanced Industrial Science and Technology Tsukuba Japan; ^3^ Department of Physical Therapy and Rehabilitation Science, Carver College of Medicine University of Iowa Iowa City Iowa USA; ^4^ Abboud Cardiovascular Research Center University of Iowa Iowa City Iowa USA; ^5^ Fraternal Order of Eagles Diabetes Research Center University of Iowa Iowa City Iowa USA

**Keywords:** cerebral artery, endothelial function, exercise, hypercapnia, intermittent hypoxia, internal carotid artery

## Abstract

The endothelium plays a pivotal role in regulating cerebrovascular blood flow, and its dysfunction increases the risk of cerebrovascular disease. Endothelial shear stress, a primary mechanical stimulus for endothelial nitric oxide production, is a key modulator of vascular adaptation. In recent years, transient hypercapnia‐induced flow‐mediated dilation of the internal carotid artery (ICA‐FMD) has emerged as a valuable in vivo approach for assessing cerebrovascular endothelial function in humans. This review first synthesizes methodological advances in ICA‐FMD assessment, emphasizing the importance of transient carbon dioxide (CO_2_) inhalation, normalizing ICA‐FMD to the shear stress, and consideration of unique ICA haemodynamics. Second, it consolidates mechanistic insights and conditions for improving ICA‐FMD, elucidating effective and ineffective strategies. Intermittent hypoxia‐induced increases in shear stress improve ICA dilatory response, underscoring the pivotal role of shear rate. Although ICA blood flow during exercise has been extensively studied, data on shear rate during exercise are limited. Moderate‐intensity leg cycling that avoids hyperventilation and elevates end‐tidal CO_2_ partial pressure increases ICA shear rate and augments post‐exercise ICA‐FMD, whereas higher‐intensity exercise or small‐muscle exercise fails to produce similar benefits. These observations suggest that a threshold shear stimulus may be required for post‐exercise improvements in ICA‐FMD. Future research should establish standardized methodologies, define the shear stimulus threshold, elucidate the time course of vascular adaptations, and extend investigations to populations at elevated cerebrovascular risk. Translating these mechanistic insights into clinical strategies has the potential to optimize cerebrovascular endothelial function and thereby contribute to the prevention of cerebrovascular diseases.

## Abstract (highlights)

- **What is the topic of this review?**
  This review explores optimizing cerebrovascular endothelial function via shear stress modulation, integrating current assessment methodologies and discussing interventions to improve flow‐mediated dilation of the internal carotid artery (ICA‐FMD), an index of cerebrovascular endothelium‐dependent dilation, with primary emphasis on exercise.
- **What advances does it highlight?**
  This article highlights how to evaluate ICA‐FMD in humans and what potential interventions work and do not work for improving ICA‐FMD. Findings from previous studies underscore the importance of arterial CO_2_ control and support the potential relevance of a threshold‐shear concept for improving ICA‐FMD.

## 1 BACKGROUND

The endothelial cells lining the intimal layer of blood vessels play a crucial role in maintaining vascular homeostasis by releasing various vasoactive substances, including nitric oxide (NO) (Furchgott & Zawadzki, 1980; Moncada & Higgs, 1993). It is well established that endothelial dysfunction in peripheral vessels contributes to the pathogenesis of numerous cardiovascular and metabolic diseases, including hypertension, coronary artery disease, heart failure and type 2 diabetes (Deanfield et al., 2007). Similarly, cerebral endothelial dysfunction has been increasingly recognized as a key contributor to the development and progression of cerebrovascular diseases. Impaired cerebrovascular endothelial function has been implicated in an elevated risk of cognitive decline, vascular dementia and Alzheimer's disease (Wang et al., 2018). In this line, the endothelium‐derived NO exerts a profound influence on cerebrovascular tone and cerebral blood flow (Andresen et al., 2006; Faraci, 1992). Experimental evidence supports this association, as animals with endothelial nitric oxide synthase (eNOS) deficiency exhibit aggravated cognitive impairment and neurovascular damage in models of both vascular dementia and Alzheimer's disease, while treatment with an NO donor has been shown to ameliorate these deficits (Ahmed et al., 2022; Allerton et al., 2024; An et al., 2021). These findings underscore the crucial role of endothelial function in preserving cerebrovascular and cognitive health. Endothelial function exhibits notable heterogeneity across different vascular beds (Aird, 2007). Within this context, the cerebral endothelium plays a uniquely specialized role, serving as a fundamental component of the blood–brain barrier and contributing to the tightly regulated neurovascular environment (Daneman & Prat, 2015). Given the critical role of cerebrovascular endothelial function in maintaining cerebrovascular health (Toda et al., 2009), strategies aimed at preserving or restoring endothelial function have garnered significant attention. Interventions that enhance endothelial NO bioavailability and improve vascular reactivity may offer promising avenues for preventing or mitigating cerebrovascular diseases. The following sections outline current methodologies for assessing cerebrovascular endothelial function and explore targeted interventions, particularly those leveraging shear stress.

## 2 METHODOLOGIES FOR ASSESSING CEREBROVASCULAR ENDOTHELIAL FUNCTION IN HUMANS

Cerebrovascular endothelial function plays a fundamental role in maintaining cerebrovascular health, yet its direct assessment in humans has historically posed significant challenges. Various pharmacological methods have been used to assess cerebrovascular endothelial function in humans (Stevenson et al., 2010). Traditionally, these approaches included measuring middle cerebral artery (MCA) blood velocity or regional cerebral blood flow using methods such as transcranial Doppler or xenon‐enhanced CT following intravenous administration of endothelium‐dependent vasodilators (i.e., L‐arginine or acetazolamide). Pretnar‐Oblak et al. reported attenuated increases in MCA velocity in response to intravenous L‐arginine infusion in patients with lacunar cerebral infarctions (Pretnar‐Oblak et al., 2006), hypertension (Pretnar‐Oblak et al., 2007) and migraine (Pretnar‐Oblak, 2014) compared with healthy individuals. These attenuated responses may reflect impaired NO‐mediated endothelial function in the cerebral circulation. Despite their clinical utility, these methods lack real‐time shear‐mediated dilatory responses and/or involve invasive procedures, thus limiting their utility in dynamic physiological assessments, specifically for cerebral endothelial function.

In peripheral arteries, flow‐mediated dilation (FMD) is a widely accepted non‐invasive method to assess endothelial function through shear stress‐induced vasodilation (Thijssen et al., 2011). This technique evaluates NO‐mediated vasodilation by inducing a transient increase in shear stress following 5 min of distal ischemia and calculates the percentage increase in arterial diameter from baseline (Celermajer et al., 1992). FMD is nearly abolished by the NO synthase inhibitor *N* ^G^‐monomethyl L‐arginine (L‐NMMA), confirming its NO dependence (Green et al., 2011). Building on this concept, a breakthrough was achieved by Carter et al., who introduced a novel approach to assess cerebrovascular endothelial function using carbon dioxide (CO_2_) inhalation to increase cerebral blood flow, while measuring shear‐mediated dilation of the internal carotid artery (ICA) via Doppler ultrasound (Carter et al., 2016; Figure 1). Notably, a 3‐min CO_2_ inhalation elicited an initial increase in MCA velocity, followed by a delayed ICA dilation, suggesting a shear‐mediated response rather than a direct vasodilatory effect of CO_2_. Moreover, they found a strong association between change in shear and diameter of the ICA. Given that FMD is influenced by downstream vascular resistance (Pyke et al., 2008) and that the ICA perfuses approximately 70% of the cerebral blood flow (Schoning et al., 1994), it is reasonable to assume that ICA‐FMD reflects cerebrovascular endothelial function. This method was further refined by Hoiland et al., who demonstrated that a 30‐s CO_2_ inhalation protocol minimizes changes in blood pressure, thereby eliminating the confounding effects of CO_2_‐induced pressor responses on the ICA‐FMD (Hoiland et al., 2017). Moreover, L‐NMMA infusion acutely attenuated ICA‐FMD induced by transient hypercapnia (Figure 2c) but not ICA dilation induced by steady‐state hypercapnia (Figure 2d), despite both methods of hypercapnia increasing cerebrovascular endothelial NO production that was subsequently attenuated by l‐NMMA (Figures 2e, f) (Hoiland et al., 2022). These results suggest that ICA dilation is partly NO‐dependent when induced by transient hypercapnia, but not when induced by steady‐state hypercapnia. Extending the findings, our group recently demonstrated that the dilatory responsiveness of the ICA to shear stress is consistent across varying levels of transient hypercapnia (Sakamoto et al., 2025). In this study, 30‐s hypercapnic challenges were conducted, increasing end‐tidal CO_2_ partial pressure ($P_{\mathrm{ETC}O_{2}}$) by 6, 9 and 12 mmHg. The total shear stimulus was quantified as the shear rate area under the curve from the onset of CO_2_ inhalation to peak dilation, including and excluding baseline values ([SR_AUC_] and delta SR_AUC_ [_D_SR_AUC_], respectively) (Figure 3). Consequently, ICA‐FMD was greater with increasing target $P_{\mathrm{ETC}O_{2}}$. Notably, ICA dilation was positively associated with _D_SR_AUC_ but not with SR_AUC_. Accordingly, when normalized to _D_SR_AUC_, corrected ICA‐FMD was comparable across the three hypercapnia conditions, whereas normalization by SR_AUC_ did not yield consistent values. These findings highlight the necessity of accounting for baseline shear rate and using net increase in shear rate (_D_SR_AUC_) when interpreting ICA‐FMD across varying stimuli. Although SR_AUC_ is widely used for normalizing peripheral artery FMD responses (Thijssen et al., 2011), the unique haemodynamics of the ICA may require an alternative approach to accurately interpret ICA‐FMD. This discrepancy likely reflects the ICA's higher baseline shear rate and the relatively modest increase in shear stimulus during CO_2_ inhalation. Supporting this, Carr et al. (2020) compared endothelium‐dependent vasodilator responses between the ICA and brachial artery in young adults (Carr et al., 2020) and reported that resting shear rate was approximately 90 s^−1^ in the brachial artery and 380 s^−1^ in the ICA. Following their respective stimuli, reactive hyperaemia for the brachial artery and 30 s of hypercapnia for the ICA, the relative increase in shear rate exceeded 800% in the brachial artery but was only ∼20% in the ICA. These findings further emphasize the importance of accounting for net shear rate increase (_D_SR_AUC_) when interpreting ICA‐FMD responses. Furthermore, as a potential avenue for future research, a baseline shear rate‐adjusted SR_AUC_ method has been proposed in the peripheral arteries (Nowicki et al., 2020). Specifically, the adjusted SR_AUC_ is calculated by dividing the baseline shear rate (the 10‐s baseline SR_AUC_ before cuff occlusion) by the conventional post‐occlusive SR_AUC_, and this adjusted value is then used to normalize FMD. This approach was reported to improve the sensitivity and specificity for detecting coronary artery disease. Although such methods have not been applied in the ICA‐FMD, they may be worth exploring, particularly if they could be effective for disease detection and risk prediction. When comparing the ICA with peripheral arteries, Carr et al. (2020) provided compelling evidence that endothelium‐dependent and ‐independent vasodilator responses are not significantly correlated between the ICA and the brachial artery. Additionally, ICA‐FMD is unrelated to cerebrovascular reactivity measures such as MCA velocity and ICA blood flow responses to CO_2_. Collectively, the evidence supports the use of vessel‐specific approaches for assessing cerebrovascular endothelial health, rather than extrapolating from peripheral vascular function or CO_2_‐mediated reactivity.

**FIGURE 1:**
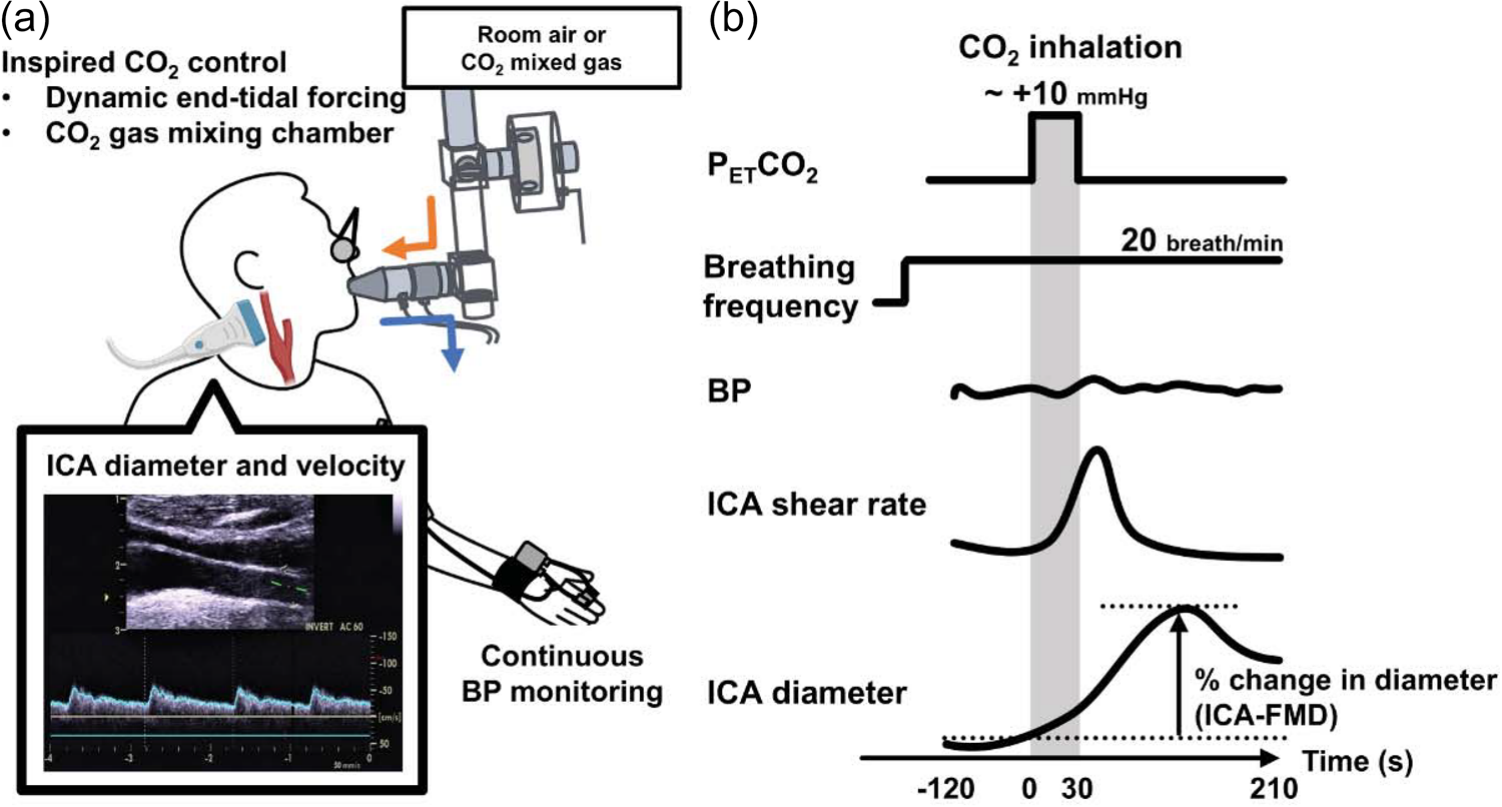
Methodology for measuring transient hypercapnia‐induced ICA‐FMD. (a) Experimental setting of the transient hypercapnia test. Partial pressure of end‐tidal CO_2_ ($P_{\mathrm{ETC}O_{2}}$) is controlled using a dynamic end‐tidal forcing system (Hoiland et al., 2022, 2017; Carr et al., 2020, 2022) or a mixing chamber (Saito et al., 2023; Sakamoto et al., 2024, 2025; Walsh et al., 2025). Diameter and blood velocity of the internal carotid artery (ICA) are measured using Doppler ultrasound. Blood pressure (BP) is continuously monitored by finger photoplethysmography. (b) Changes in cardiorespiratory parameters. Thirty seconds of CO_2_ inhalation elevates $P_{\mathrm{ETC}O_{2}}$ to ∼+10 mmHg from the pre‐hypercapnia level. Breathing frequency is maintained at 20 breaths per minute for quicker achievement of the target $P_{\mathrm{ETC}O_{2}}$ level. ICA flow‐mediated dilation (ICA‐FMD) is expressed as the percentage change in ICA diameter from baseline values.

**FIGURE 2:**
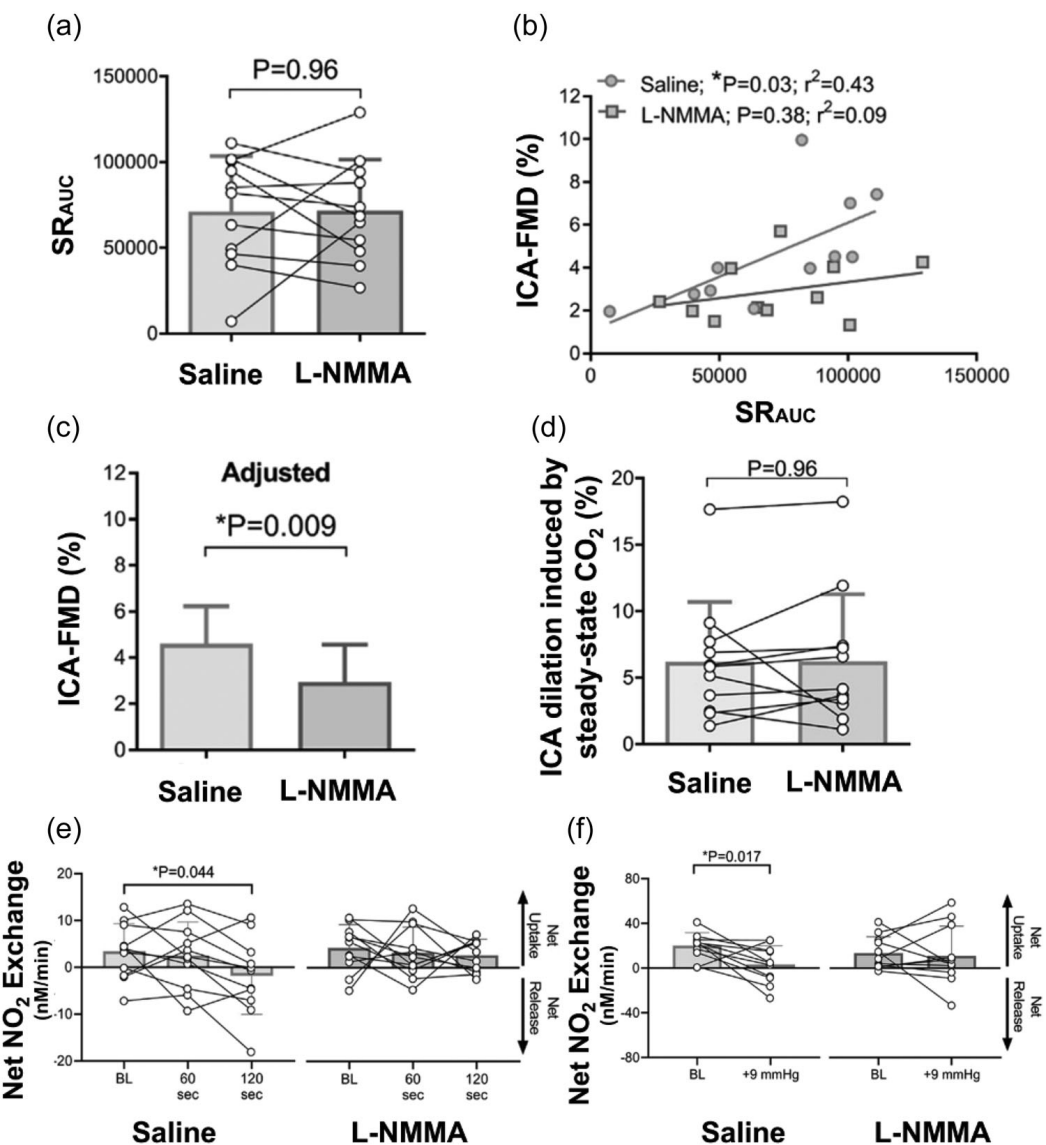
Effects of nitric oxide synthase inhibition on ICA‐FMD. (a) Shear rate area under the curve (SR_AUC_) from the onset of hypercapnia to ICA peak dilation. (b) Positive correlation between SR_AUC_ and ICA flow‐mediated dilation (ICA‐FMD) during the saline trial, which was not evident with l‐NMMA infusion. (c) ICA‐FMD, corrected by baseline diameter and SR_AUC_ as covariates in linear mixed model analyses, was attenuated during l‐NMMA infusion compared to saline. (d) ICA dilation induced by steady‐state hypercapnia (stepwise increases in partial pressure of end‐tidal CO_2_ by +4.5 and +9 mmHg above baseline, each maintained for 5 min) was unchanged during l‐NMMA infusion compared to saline. (e, f) Net exchange of NO_2_^−^ (uptake/release) during transient (e) and steady‐state (f) hypercapnia. Data in all panels except (b) are presented as means ± SD, with lines connecting paired individual data points. *Significant difference, *P* < 0.05. (Modified from Hoiland et al., 2022)

**FIGURE 3:**
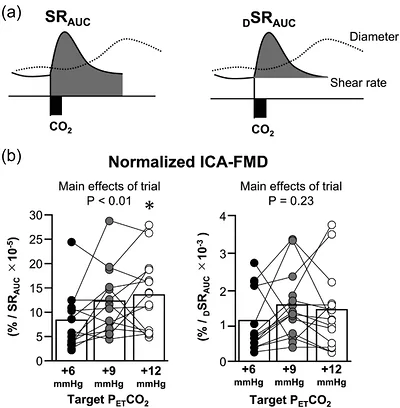
Between‐trial differences in ICA‐FMD persist when normalized using SR_AUC_ but disappear when normalized using _D_SR_AUC_ across different hypercapnia levels. (a) Change in shear rate and diameter of the internal carotid artery (ICA). Grey areas are the shear rate area under the curve from the onset of CO_2_ inhalation to peak dilation, including and excluding baseline values [(SR_AUC_) at left and delta SR_AUC_ (_D_SR_AUC_) at right, respectively]. (b) ICA flow‐mediated dilation (ICA‐FMD) normalized by SR_AUC_ (left) and _D_SR_AUC_ (right). Mean (bars) and individual data (circles). *P < 0.05 versus +6 mmHg. (Modified from Sakamoto et al. 2025)

Using this CO_2_ inhalation technique, our group has demonstrated that ICA dilatory response, assessed using a 3‐min hypercapnic stimulus, is attenuated by sympathetic activation (Iwamoto et al., 2018a) and ageing (Iwamoto et al., 2018b). In contrast, ICA dilation induced by 3‐min hypercapnia was not attenuated by habitual cigarette smoking in young adults (Suzuki et al., 2020). Similarly, transient hypercapnia‐induced ICA‐FMD is not significantly affected by 4‐h prolonged sitting (Saito et al., 2025). Moreover, steady‐state hypercapnia‐induced ICA dilatory response fluctuates across the menstrual cycle in premenopausal women, with the highest values observed during the late follicular phase (higher oestradiol) and progressively declines with advancing menopausal stage. Notably, lower serum oestradiol levels during the menstrual cycle and across menopause stages are associated with attenuated ICA dilatory response to steady‐state CO_2_ (Iwamoto et al., 2021). Collectively, ICA dilatory responses induced by steady‐state hypercapnia have demonstrated sensitivity to several physiological modifiers, including sympathetic activation, ageing and oestrogen deficiency, supporting their physiological validity. In contrast, ICA dilation induced by transient and steady‐state hypercapnia appears to be relatively unaffected by behavioural stressors such as prolonged sitting and smoking, both of which are known to impair peripheral FMD (Haptonstall et al., 2020; Taylor et al., 2022), suggesting the presence of unique regulatory or protective mechanisms in cerebral circulation. Given the methodological shift from steady‐state to transient hypercapnia in evaluating cerebrovascular endothelial function, it is desirable that the ICA‐FMD technique will become further established in the future. To our knowledge, there is currently no published data reporting the reproducibility of ICA‐FMD assessed using transient hypercapnia. Regarding reliability, recent studies have reported coefficients of variation (CV) for ICA diameter during transient ICA‐FMD. For example, the within‐day CV for ICA diameter was 1.5% (Hoiland et al., 2017) and 1.66 ± 0.95% (Sakamoto et al., 2025), whereas the between‐day CV was 2.9 ± 1.3% (Sakamoto et al., 2024). In addition, Carr et al. reported CVs for resting ICA variables as follows: diameter = 3.0%, velocity = 9.6% and flow = 11.7% (Carr et al., 2022). These findings indicate that ICA variables can be assessed with high reliability. Advancing ICA‐FMD as a reliable research and clinical tool requires continued methodological refinement, including the development of standardized protocols, validation of reproducibility, establishment of normative reference ranges in healthy individuals, and identification of physiological factors that may influence the measure. Ultimately, such progress may support the use of ICA‐FMD as a prognostic marker, reinforcing its value in cerebrovascular risk stratification and preventive health strategies.

## 3 SHEAR STRESS‐MEDIATED ENDOTHELIAL ADAPTATION

In peripheral vessels, compelling evidence has established shear stress as a key modulator of endothelial function. Lower limb exercise increases shear rate and enhances brachial artery FMD; however, this favourable effect is abolished in a cuffed arm where blood flow is locally restricted, despite identical systemic conditions (Birk et al., 2012; Tinken et al., 2010). In this context, elevated shear stress is recognized as a key haemodynamic signal underlying endothelial adaptation. Furthermore, direct evidence in humans demonstrated that elevated shear stress in the radial or brachial arteries induced by handgrip exercise increases endothelial NO synthase Ser1177 phosphorylation in endothelial cells (Casey et al., 2017; Park et al., 2019; Tryfonos et al., 2023). This post‐translational modification confirms that muscle contraction‐induced increases in shear stress led to functional activation of eNOS at the cellular level in the peripheral arteries.

The established role of shear stress in modulating peripheral endothelial function raises the question of whether similar mechanisms operate in the cerebral circulation. Experimental work with isolated cerebral endothelial cells of animals demonstrated a progressive decline in NO production during experimental cessation of perfusate flow, followed by a time‐dependent increase in NO levels during reperfusion (Krizanac‐Bengez et al., 2003). Consistent with this, an in vitro model using human cerebral endothelial cells demonstrated that NO concentration increased in response to elevated shear stress (Mashour & Boock, 1999). Furthermore, in isolated animal cerebral arteries, increasing intraluminal flow elicited shear‐mediated dilation, which was abolished by endothelial denudation or by inhibition of endothelial NO synthase with *N* ^ω^‐nitro‐ L‐arginine methyl ester (L‐NAME) (Cosic et al., 2016; Drouin & Thorin, 2009). These results collectively support the concept that cerebral endothelial function is also regulated in a shear‐dependent manner. Building upon this foundation, recent studies have begun to explore whether targeted interventions can enhance cerebrovascular endothelial function through shear stress‐mediated mechanisms. In this context, acute cyclic intermittent hypoxia (IH) has been shown to increase MCA blood velocity in healthy adults (Liu et al., 2017). Moreover, cerebral vasodilation during hypoxia appears to be partially mediated by NO (Hoiland et al., 2023; Van Mil et al., 2002), and IH training has been reported to elevate circulating markers of NO bioavailability (Lyamina et al., 2011). Based on this rationale, we investigated whether IH‐induced increases in shear rate could enhance cerebrovascular endothelial function in healthy adults (Iwamoto et al., 2020). Participants underwent five cycles of 6‐min hypoxia (target peripheral oxygen saturation ∼80%) interspersed with 4‐min normoxia. This protocol cyclically elevated ICA shear rate and significantly increased ICA dilatory response to 3‐min CO_2_ challenge following IH, whereas no changes in shear rate and ICA dilation were observed following continuous normoxia (Sham trial) (Figure 4). This finding provides the first in vivo evidence in humans that acute increases in shear stress can enhance ICA dilatory response to steady state CO_2_. In the peripheral arteries, acute post‐exercise changes in brachial FMD are related to changes in resting FMD after exercise training (Dawson et al., 2017), suggesting that acute responses may reflect chronic adaptations. However, current evidence is still limited, and a definitive conclusion has not yet been established even in the peripheral arteries. Similarly, elucidating whether an increase in the ICA dilatory response following interventions such as IH contributes to long‐term adaptations in ICA‐FMD will be an important direction for future research.

**FIGURE 4:**
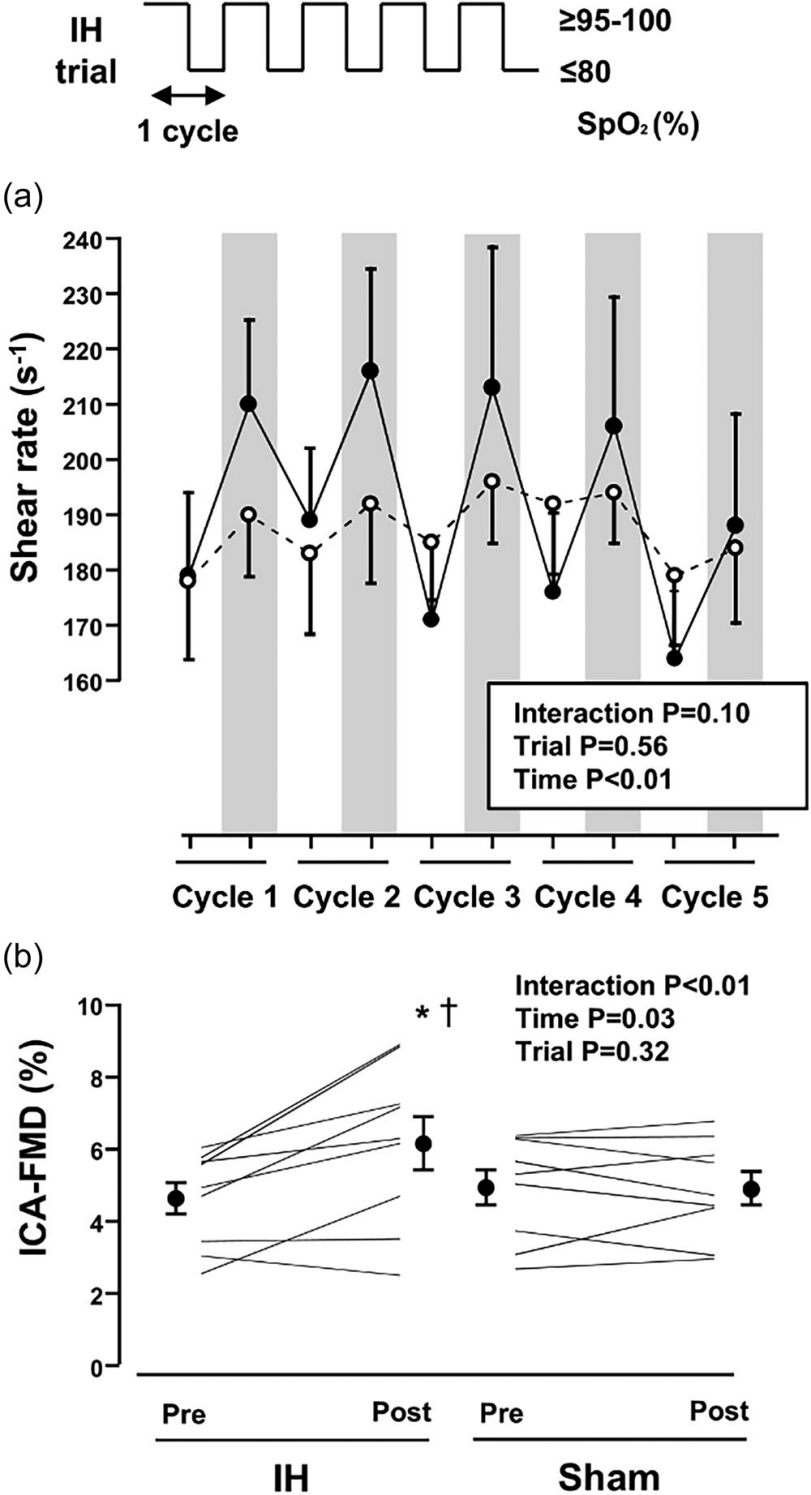
Shear rate elevation during intermittent hypoxia leads to improved post‐intervention ICA‐FMD. (a) Changes in shear rate of the internal carotid artery (ICA) during intermittent hypoxia (IH, solid line) and control normoxia (Sham, dotted line). White and grey bars represent normoxic and hypoxic phases in the IH trial, respectively. (b) Individual (lines) and group mean (circles, mean ± standard error) for ICA flow‐mediated dilation (ICA‐FMD) before and following IH and Sham trials. *P < 0.05 versus Pre in IH, †*P *< 0.05 versus Post in sham. (Modified from Iwamoto et al., 2020)

## 4 EXERCISE‐INDUCED SHEAR STRESS DYNAMICS IN THE ICA

Recognizing the influence of shear stress on cerebrovascular endothelial function, physical exercise has garnered attention as a practical and physiologically grounded approach to increasing cerebral blood flow and shear rate. Sato et al. demonstrated that ICA blood flow progressively increases with exercise intensity up to approximately 60% of peak oxygen uptake (${\dot{V}}_{O_{2}\mathrm{peak}}$), but declines and returns to baseline at 80% ${\dot{V}}_{O_{2}\mathrm{peak}}$ during leg cycling (Sato et al., 2011). The reduction in ICA blood flow at higher intensity exercise (80% ${\dot{V}}_{O_{2}\mathrm{peak}}$) closely mirrored a decline in $P_{\mathrm{ETC}O_{2}}$. Moreover, they observed a substantial rise in external carotid artery (ECA) blood flow from moderate to high intensities, which was strongly and inversely correlated with ICA blood flow. These findings suggest that redistribution of blood flow toward the ECA may contribute to the decrease in ICA blood flow during higher‐intensity exercise. This shift likely reflects thermoregulatory demands, as ECA blood flow changes were significantly associated with increases in forehead cutaneous vascular conductance throughout the exercise session. A comprehensive review by Smith and Ainslie also reported that MCA velocity during aerobic exercise exhibits an inverted U‐shaped response, with elevations during low‐ to moderate‐intensity exercise followed by reductions at higher‐intensity due to hyperventilation‐induced decreases in arterial carbon dioxide pressure (Smith & Ainslie, 2017), which lead to cerebral vasoconstriction (Ogoh & Ainslie, 2009a, 2009b). While dynamic exercise has been shown to alter ICA blood flow depending on exercise intensity, it remains unclear how these changes influence ICA *shear rate* and whether they elicit improvements in cerebrovascular endothelial function. Importantly, alterations in blood flow do not always correspond to changes in shear rate. Since shear rate is influenced by both blood flow and vessel diameter, a reduction in diameter can augment shear rate even when blood flow is unchanged. Accordingly, direct quantification of shear rate is critical for elucidating endothelial stimuli and their impact on vascular function.

Regarding the ICA shear rate during exercise, Smith et al. (2019) reported interesting findings, with particular attention to $P_{\mathrm{ETC}O_{2}}$ levels. In their study, healthy young adults completed three 30‐min interventions: (1) mild hypercapnia, (2) leg cycling at 60% HR reserve, and (3) resting control matched for duration. Both hypercapnia and moderate‐intensity exercise induced similar increases in $P_{\mathrm{ETC}O_{2}}$. These changes were accompanied by comparable elevations in ICA shear rate from baseline, as well as similar vasodilation magnitudes (ICA: 5.3 ± 0.8% dilation for hypercapnia vs. 4.7 ± 0.7% for exercise). Although they reported an increase in ICA shear rate during moderate‐intensity leg cycling, our group observed contrasting results (Sakamoto et al., 2021). In our study, participants completed two separate 30‐min leg cycling protocols: moderate‐intensity exercise [65 ± 5% of age‐predicted maximal heart rate (HR_max_)] and high‐intensity exercise (85 ± 5% HR_max_). However, neither exercise session elevated ICA shear rate, and ICA conductance decreased during high‐intensity exercise. $P_{\mathrm{ETC}O_{2}}$ during exercise remained unchanged at moderate‐intensity but decreased below resting levels during high‐intensity exercise. These findings suggest that the effect of exercise on ICA shear rate may depend on exercise intensity. Indeed, Moir et al. (2024) recently reported that, although not during exercise, post‐exercise ICA shear stress exhibits different responses depending on exercise intensity. In their study, healthy adults completed three treadmill protocols: 30% or 70% of maximal oxygen uptake (${\dot{V}}_{O_{2}\max}$) for 30 min, and a shorter 70% ${\dot{V}}_{O_{2}\max}$ matched for energy expenditure (energy‐equivalent, EqEE). Both post‐exercise ICA blood flow and shear stress (i.e., shear rate adjusted for blood viscosity) were higher after the longer 70% ${\dot{V}}_{O_{2}\max}$ session than after the 30% ${\dot{V}}_{O_{2}\max}$ session. In the 70% ${\dot{V}}_{O_{2}\max}$ condition, the full trial increased blood flow more than EqEE, but shear stress showed no difference between trials. Taken together, these results suggest that discrepancies in ICA shear rate across studies may partly arise from differences in exercise intensity and the accompanying changes in $P_{\mathrm{ETC}O_{2}}$ during exercise. Understanding the interaction between these factors will be crucial for predicting cerebrovascular responses to exercise.

Consistent with this notion, both our study (Sakamoto et al., 2021) and that of Smith et al. (2019) employed moderate‐intensity dynamic exercise; however, ICA shear rate responses differed between the two investigations. One possible explanation for this discrepancy is the difference in $P_{\mathrm{ETC}O_{2}}$ responses during exercise, which may be influenced by an individual's aerobic fitness level. It has been reported that individuals with lower aerobic fitness tend to initiate hyperventilation at lower exercise intensity, which can attenuate the rise in $P_{\mathrm{ETC}O_{2}}$ typically observed during moderate‐intensity exercise (Manetta et al., 2005; Shimamoto & Komiya, 2003). The subjects in our study may have had relatively lower aerobic fitness compared to those in Smith's study (2019), which led to an earlier onset of hyperventilation, thereby blunting the $P_{\mathrm{ETC}O_{2}}$ elevation and suppressing the ICA shear rate increase during exercise. This explanation is further supported by the findings of our recent study (Sakamoto et al., 2024), in which we demonstrated moderate‐intensity leg cycling at 80% of ventilatory threshold (VT) induced an increase in $P_{\mathrm{ETC}O_{2}}$ and a significant elevation in ICA shear rate throughout the 30‐min exercise session. However, when subjects were instructed to hyperventilate during exercise at the same absolute intensity, the resulting reduction in $P_{\mathrm{ETC}O_{2}}$ was accompanied by no increase in ICA shear rate during exercise. These findings highlight the critical role of $P_{\mathrm{ETC}O_{2}}$ in regulating ICA shear rate during exercise and suggest that acute aerobic exercise performed at an intensity that does not induce excessive hyperventilation and elevates $P_{\mathrm{ETC}O_{2}}$, such as slightly below the VT level, may be effective in enhancing the ICA shear stimulus during exercise.

In addition to exercise intensity, exercise modality also influences ICA shear responses. Ogoh et al. (2021) compared continuous and interval leg cycling at moderate intensity, matched for total energy expenditure. Participants cycled continuously at 80 W for 12 min or in an interval protocol consisting of three bouts of 2 min at 60 W followed by 2 min at 100 W, matching the total workload. Despite this equivalence, interval exercise elicited significantly greater increases in ICA shear rate, during both exercise and recovery. A follow‐up study by the same group also showed greater post‐exercise ICA shear rate after interval compared to continuous exercise (Walsh et al., 2025). These findings suggest that acute interval exercise can amplify shear stimuli in the cerebral circulation. In summary, current evidence indicates that both exercise intensity and exercise modality appear to influence ICA shear stress primarily through alterations in $P_{\mathrm{ETC}O_{2}}$ during exercise. These interactions determine the magnitude of shear stimuli acting on the cerebrovascular endothelium. Whether exercise‐induced repeated acute increases in cerebral shear and associated vascular responses lead to functional adaptations in the cerebrovascular endothelium is an important question that deserves further elucidation.

## 5 IMPROVING CEREBROVASCULAR ENDOTHELIAL FUNCTION THROUGH EXERCISE‐INDUCED SHEAR

Given that exercise can modulate shear stress dynamics in the ICA, it is reasonable to propose that targeted exercise interventions may also induce favourable adaptations in cerebrovascular endothelial function. In support of this, in vivo animal experiments demonstrated that exercise training restored impaired eNOS‐dependent vasodilation of cerebral arteries in diabetic rats (Mayhan et al., 2011, 2004). Despite the vascular benefits of exercise in the peripheral arteries of humans being well documented, comparable evidence for the ICA remains limited. A study published in 2021 (Sakamoto et al., 2021) was the first to investigate exercise‐induced changes in ICA dilatory response to steady‐state hypercapnia in humans. In this study, ICA dilation was assessed in healthy young men using a 3‐min hypercapnic stimulus at baseline, and at 5 and 60 min following 30 min of leg cycling at moderate intensity (65 ± 5% of HR_max_) and high intensity (85 ± 5% HR_max_). Neither exercise condition elicited an increase in ICA shear rate during exercise. Consequently, ICA dilatory response to steady‐state hypercapnia remained unchanged after moderate‐intensity exercise, whereas it significantly decreased 5 min after high‐intensity exercise and returned to baseline by 60 min post‐exercise. These findings suggest that exercise failing to adequately enhance ICA shear rate is unlikely to improve ICA dilatory response. Moreover, post‐exercise changes in ICA dilation after higher‐intensity exercise appear to follow a biphasic pattern, similar to that observed in peripheral arteries (Dawson et al., 2013). In the peripheral artery, the acute reduction in FMD following high‐intensity exercise may be attributed to increased sympathetic nerve activity (Atkinson et al., 2015) and elevated oxidative stress (Johnson et al., 2012). Given that ICA dilatory response to steady‐state hypercapnia is also attenuated by sympathetic stimulation (Iwamoto et al., 2018a), and that oxidative stress impairs cerebrovascular endothelial function (Freeman & Keller, 2012), a similar mechanism may contribute to the transient reduction in ICA dilation observed after high‐intensity exercise. In addition to aerobic exercise involving large muscle groups, small‐muscle exercises such as handgrip exercise have also been investigated for their effects on cerebrovascular function and cognition. In a study by Saito et al. (2023), young adults performed interval handgrip exercise consisting of four sets of 2‐min contractions at 25% of maximal voluntary contraction, interspersed with 3‐min rest periods. Handgrip exercise elicited no increase in ICA shear rate; accordingly, transient hypercapnia‐induced ICA‐FMD remained unchanged at 5 and 60 min following the intervention. These findings suggest that small‐muscle exercises may be insufficient to elicit the shear stimulus necessary to improve cerebrovascular endothelial function (Saito et al., 2023).

Building on the premise that shear stress serves as a critical stimulus for vascular adaptation, a key question arises: can exercise that effectively increases ICA shear rate lead to improvements in cerebrovascular endothelial function, as assessed by ICA‐FMD? As previously discussed, increases in $P_{\mathrm{ETC}O_{2}}$ during exercise appear to be a key determinant of elevated ICA shear rate. To directly test this hypothesis, we conducted a study in which young adults completed four exercise interventions designed to differentially manipulate ICA shear rate during aerobic exercise (Sakamoto et al., 2024) (Figure 5). All sessions were performed at an intensity corresponding to 80% of VT. Subjects either breathed spontaneously (Ex_SB_, SR increase), hyperventilated without added CO_2_ (Ex_HV_, no increase in SR), or hyperventilated with the addition of CO_2_ to inspiratory air (Ex_HV+CO2_, restoration of SR increase). A non‐exercise sitting rest condition (CON) was also included. ICA‐FMD was assessed before and 10 min after each 30‐min intervention using a 30‐s hypercapnia challenge. When ICA‐FMD was normalized to the net increase in shear stimulus above baseline (_D_SR_AUC_), a significant post‐exercise increase was observed in the Ex_SB_ trial, where ICA shear rate was elevated during exercise. Notably, this enhancement in ICA‐FMD was abolished in the Ex_HV_ trial, where hyperventilation suppressed the shear rate response. In contrast, when hyperventilation was accompanied by CO_2_ inhalation (Ex_HV+CO2_), thereby restoring the shear rate increase, the post‐exercise augmentation in ICA‐FMD reappeared. No significant change was observed following the 30‐min sitting rest. Collectively, these findings underscore the potential of aerobic exercise to improve ICA‐FMD when it elicits an adequate shear stimulus, which is modulated in part by arterial CO_2_ retention. This provides a promising physiological foundation for developing targeted exercise strategies aimed at improving cerebrovascular endothelial health. However, future studies are warranted to determine whether these acute effects translate into long‐term adaptations, and to clarify the duration and frequency of exercise training required to achieve sustained benefits. On the other hand, Walsh et al. recently reported that ICA‐FMD did not increase 15 or 45 min after an interval cycling protocol previously shown to elicit approximately a 30% increase in ICA shear rate during exercise (Ogoh et al., 2021; Walsh et al., 2025). In contrast, our study demonstrated a beneficial effect of exercise on ICA‐FMD, reporting that ICA shear rate increased by >60% from baseline during exercise (Sakamoto et al., 2024). These observations suggest that a certain threshold of shear rate increase may be necessary to elicit favourable adaptations in ICA‐FMD, warranting further research to define this threshold for exercise‐induced improvements in cerebrovascular endothelial function.

**FIGURE 5:**
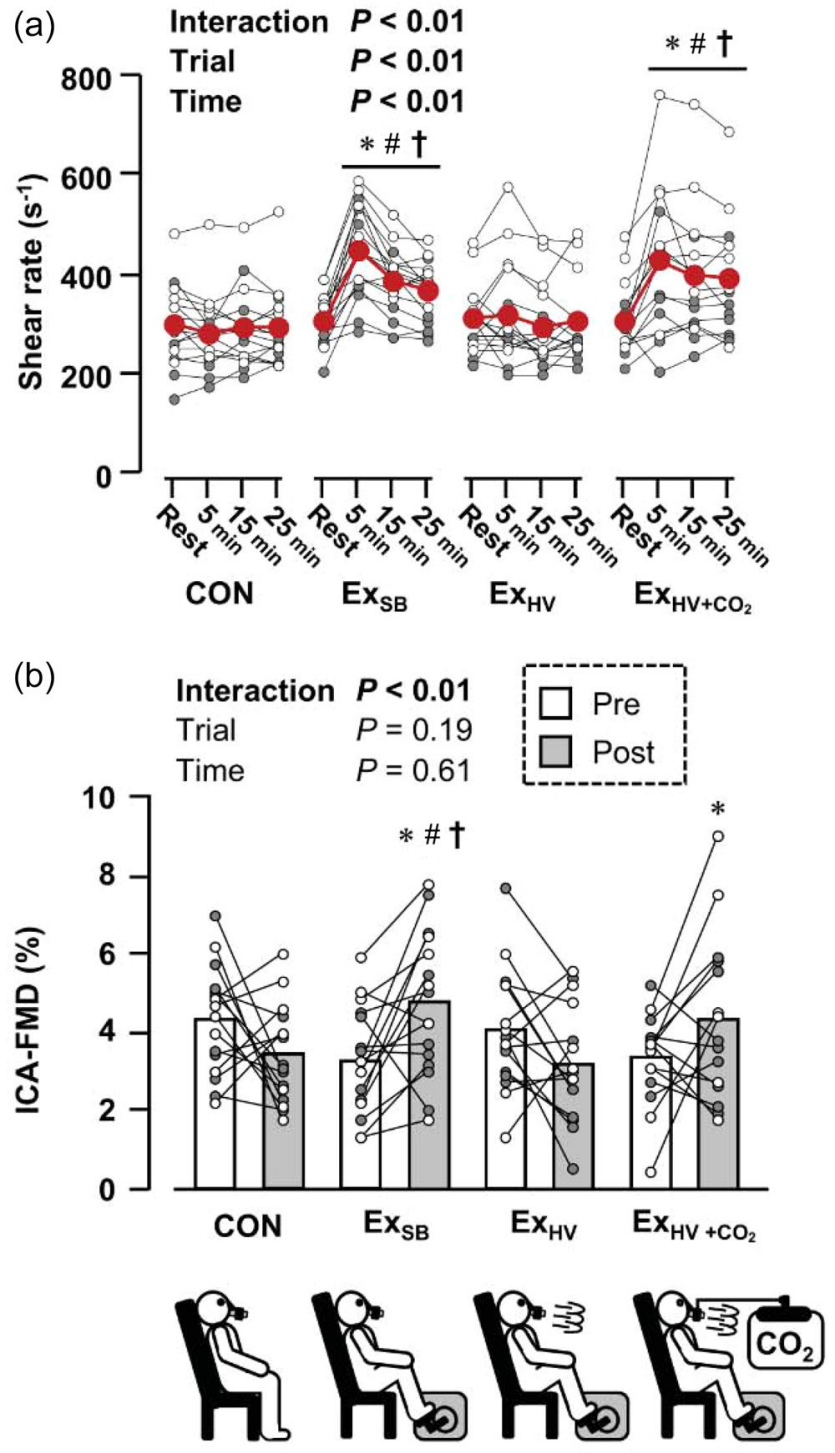
Shear rate during exercise and post‐exercise ICA‐FMD. (a) Shear rate. Red circles represent the mean values, and individual data for men (grey) and women (white) are indicated with circles. (b) Flow‐mediated dilation of the internal carotid artery (ICA‐FMD). There was no change in ICA shear rate or ICA‐FMD during 30 min of sitting rest (CON). During spontaneous breathing at 80% of the ventilatory threshold (Ex_SB_), partial pressure of end‐tidal CO_2_ ($P_{\mathrm{ETC}O_{2}}$) and shear rate increased during exercise, and ICA‐FMD improved after exercise. However, at the same intensity, hyperventilation (Ex_HV_) caused a decrease in $P_{\mathrm{ETC}O_{2}}$, a reduction in shear rate during exercise, and no improvement in ICA‐FMD. Even with hyperventilation, when CO_2_ was administered to restore $P_{\mathrm{ETC}O_{2}}$ to the Ex_SB_ level (Ex_HV+CO2_), shear rate increased and ICA‐FMD improved. *P < 0.05 versus Rest (a) and Pre (b), #*P* < 0.05 versus CON, †*P* < 0.05 versus Ex_HV_. (Modified from Sakamoto et al. 2024)

## 6 SUMMARY AND FUTURE DIRECTIONS

Cerebrovascular endothelial function is essential for the maintenance of cerebral blood flow and cognitive function. Accumulating evidence indicates that transient CO_2_‐induced flow/shear‐mediated dilation of the ICA (ICA‐FMD) serves as a reliable approach for assessing cerebrovascular endothelial function in humans. It has been established that using a transient (i.e., 30 s) CO_2_ inhalation and correcting dilation for the net increase in shear stimulus above baseline (_D_SR_AUC_) are critical to ensuring the validity of ICA‐FMD. Future research aimed at promoting the broader implementation of ICA‐FMD in clinical and research settings should focus on the standardization of measurement protocols and the establishment of normative reference values. Expanding evidence in populations at elevated risk for cerebrovascular disease, particularly in clinical cohorts, will also be important for advancing the translation of ICA‐FMD into effective clinical applications.

Since the cerebral arteries exhibit shear‐mediated and partly NO‐mediated dilation, interventions that effectively enhance shear stress represent a logical strategy to improve cerebrovascular endothelial function. Acute increases in shear stress induced by intermittent hypoxia enhance the ICA dilatory response to steady‐state hypercapnia. Similarly, elevated $P_{\mathrm{ETC}O_{2}}$ during exercise have been shown to improve ICA‐FMD. Exercise is a promising strategy to enhance ICA shear stress; however, its effects may depend on the intensity and exercise mode, as well as the ventilatory response during exercise. Along these lines, moderate‐intensity leg cycling without inducing hyperventilation has been shown to improve post‐exercise ICA‐FMD in healthy young adults, whereas higher‐intensity leg exercise or small‐muscle mass exercise does not appear to provide such benefits. Clarifying the shear rate threshold that elicits ICA‐FMD enhancement will be pivotal for guiding the design of targeted interventions to promote cerebrovascular endothelial function. Moreover, elucidating the time course and durability of training‐induced vascular adaptations will be an important step in advancing this field (Figure 6). To facilitate clinical application, it will be essential to determine whether improvements in ICA‐FMD reduce the risk of cerebrovascular diseases and whether ICA‐FMD predicts future cerebrovascular events. Clarifying these aspects will help establish its utility for assessing the efficacy of preventive and therapeutic strategies, as these efforts will ultimately contribute to the maintenance of cerebrovascular health.

**FIGURE 6:**
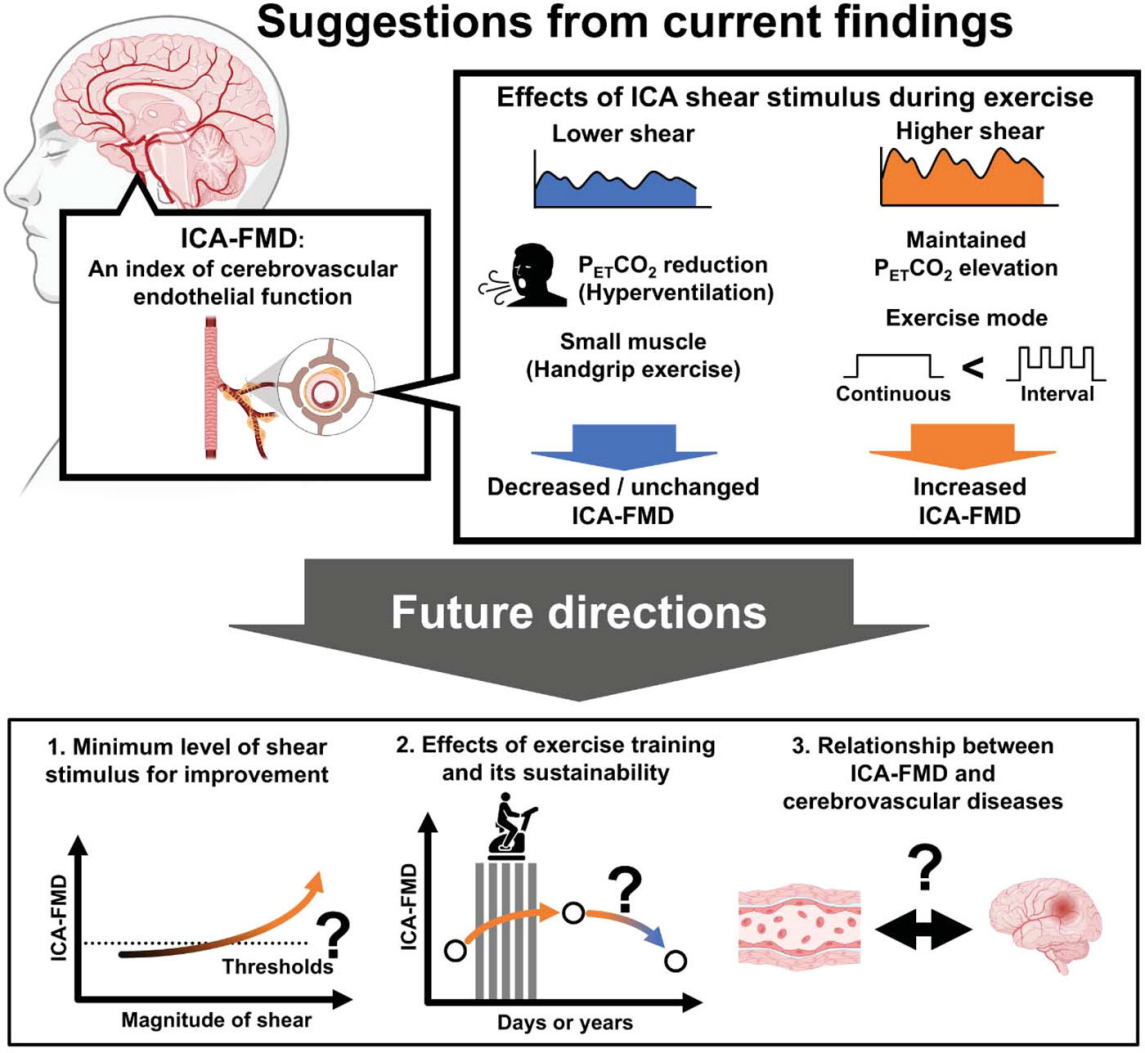
Suggestions from current findings and future directions. Current evidence has identified factors influencing the effect of exercise‐induced increases in ICA shear stimulus on post‐exercise flow‐mediated dilation of the internal carotid artery (ICA‐FMD), delineating what potential interventions work and do not work. Future research should address: (1) the minimum shear stimulus required to improve cerebrovascular endothelial function; (2) the effects of exercise training and the persistence of its benefits; and (3) the relationship between ICA‐FMD and cerebrovascular disease.

## AUTHOR CONTRIBUTIONS

In this study, Erika Iwamoto was primarily responsible for research, concept development, and preparation of the text, figures, and references. Rintaro Sakamoto contributed to the preparation and editing of the figures and text in this manuscript. Darren P. Casey was responsible for the overall editing of the text and figures, as well as for concept development. All authors approved the final version of the manuscript and agree to be accountable for all aspects of the work, ensuring that any questions related to the accuracy or integrity of any part of the work are appropriately investigated and resolved. All persons designated as authors qualify for authorship, and all those who qualify for authorship are listed.

## CONFLICT OF INTEREST

None declared.
